# Association Between Female Reproductive Factors and Laryngopharyngeal Reflux: A National Population-Based Study

**DOI:** 10.3390/jcm15135279

**Published:** 2026-07-06

**Authors:** Kyung Hoon Park, Jung Ho Park, Hong Kyu Lee, Mi Jung Kwon, Heejin Kim, Jeong Wook Kang

**Affiliations:** 1 Department of Otorhinolaryngology-Head and Neck Surgery, Hallym University Sacred Heart Hospital, Hallym University College of Medicine, Anyang 14068, Republic of Korea; pzpdlh@hallym.or.kr (K.H.P.); mir5020@hallym.or.kr (H.K.); 2 Department of Breast and Endocrine Surgery, Hallym University Sacred Heart Hospital, Hallym University College of Medicine, Anyang 14068, Republic of Korea; 3 Department of Cardiovascular Surgery, Hallym University Sacred Heart Hospital, Hallym University College of Medicine, Anyang 14068, Republic of Korea; hklee0228@hallym.or.kr; 4 Department of Pathology, Hallym University Sacred Heart Hospital, Hallym University College of Medicine, Anyang 14068, Republic of Korea; mulank99@hallym.or.kr

**Keywords:** laryngopharyngeal reflux, female hormones, reproductive history, gastroesophageal reflux, epidemiology

## Abstract

**Background:** The association between female reproductive factors and laryngopharyngeal reflux (LPR) remains poorly understood. This study investigated the relationship between reproductive history and LPR risk in women using a large nationally representative cohort. **Methods:** We conducted a nested case–control study using linked data from the Korean National Health and Nutrition Examination Survey and the National Health Insurance Service database (2007–2017). Among 57,559 participants, 1347 women diagnosed with LPR were matched 1:1 with 1347 controls without reflux disease using propensity score matching based on age, income, and education level. Multivariable logistic regression analyses were performed to evaluate the associations between reproductive factors and LPR risk after adjustment for metabolic and lifestyle variables. **Results:** Women with a first childbirth at age ≥ 27 years had a significantly higher risk of LPR than those with earlier childbirth (adjusted odds ratio [aOR] 1.33, 95% confidence interval [CI] 1.08–1.63; *p* = 0.007). A menarche-to-first-birth interval of ≥17 years was also associated with an increased risk of LPR (aOR 1.56, 95% CI 1.15–2.15; *p* = 0.005). In addition, breastfeeding for <12 months was independently associated with a higher risk of LPR (aOR 1.26, 95% CI 1.04–1.53; *p* = 0.019). These associations remained significant after adjustment for potential confounders. **Conclusions:** Female reproductive factors potentially reflecting cumulative hormonal exposure were independently associated with LPR risk in this nationally representative cohort. First delivery at age ≥ 27 years, a menarche-to-first-birth interval ≥ 17 years, and breastfeeding < 12 months were associated with LPR, suggesting that reproductive history should be considered when evaluating women with reflux-related symptoms.

## 1. Introduction

Laryngopharyngeal reflux (LPR) is a prevalent and clinically significant condition characterized by the retrograde flow of gastric contents into the larynx and pharynx, leading to symptoms such as chronic cough, throat clearing, globus sensation, and hoarseness. These symptoms are commonly evaluated using instruments such as the Reflux Symptom Index and the Reflux Finding Score [1,2]. LPR has been shown to substantially impair quality of life and impose a considerable health burden [3]. Despite its clinical importance, the diagnosis and treatment of LPR remain challenging due to nonspecific symptoms, heterogeneous clinical presentations, and the absence of universally accepted diagnostic criteria [4]. Moreover, increasing evidence suggests that LPR may exhibit distinct pathophysiological mechanisms from gastroesophageal reflux disease (GERD), supporting the concept that LPR is not merely an extraesophageal manifestation of GERD but a separate clinical entity [4].

Previous studies on LPR have primarily focused on lower esophageal sphincter dysfunction, acid exposure, and esophageal motility abnormalities as key mechanisms of reflux-related pathology [4]. However, epidemiological data indicate notable sex differences in reflux-related disorders, with a higher prevalence observed in women, particularly during the perimenopausal and post-reproductive years [5]. These findings suggest that hormonal or reproductive factors may play a role in susceptibility to reflux beyond traditional mechanical explanations. The mechanisms underlying this sex disparity are poorly understood and are not adequately explained by conventional reflux physiology alone.

Several studies have suggested that female sex hormones, including estrogen and progesterone, influence gastrointestinal motility and lower esophageal sphincter pressure, potentially predisposing women to reflux [6]. Additionally, pregnancy, menopause, and hormone replacement therapy have been associated with a higher risk of GERD, indicating that hormonal fluctuations may modulate reflux mechanisms [5]. However, little is known about the association between female reproductive history, a surrogate marker of lifetime hormonal exposure, and the risk of LPR. No large-scale population-based study has specifically investigated this relationship. Therefore, this study aimed to assess the association between reproductive factors and the risk of LPR in women using a nationally representative cohort. We hypothesized that reproductive factors reflecting cumulative hormonal exposure would be associated with an increased risk of LPR.

## 2. Materials and Methods

### 2.1. Study Design and Data Source

This nested case–control study, conducted within a retrospective claims-based cohort, used an integrated dataset derived from the Korean National Health Insurance Service (KNHIS) and the Korea Disease Control and Prevention Agency (KDCA) [7]. The dataset included participants from the Korea National Health and Nutrition Examination Survey (KNHANES), which is a nationwide population-based health survey designed to assess the health and nutritional status of the Korean population [8]. KNHANES, conducted by the KDCA, provided standardized health-examination and questionnaire data (including reproductive history, anthropometrics, and lifestyle factors), which were linked at the individual level to NHIS claims data (diagnoses, procedures, and prescriptions) used to operationalize the LPR definition. The two sources are complementary rather than identical. We extracted medical claim data of 57,559 participants who underwent KNHANES health examinations between 2007 and 2017 from the KNHIS database and constructed a longitudinal cohort by linking individual health survey data with healthcare utilization records.

### 2.2. Definition of Laryngopharyngeal Reflux

LPR was defined by the following criteria: (1) Presence of a primary or secondary diagnosis of reflux disease (International Classification of Diseases, 10th Revision [ICD-10] codes K21, K21.0, or K21.9) in combination with at least one LPR-related upper aerodigestive symptom code, including chronic laryngitis (J37), chronic cough (R05), throat discomfort/globus sensation (R09.8), dysphonia (R49.0), or pharyngitis (J31.2). (2) Evidence of laryngeal evaluation using laryngoscopy or 24-h hypopharyngeal–esophageal multichannel intraluminal impedance–pH monitoring, performed within one month of diagnosis. Healthcare utilization history and prescription data were sorted chronologically for each subject to confirm diagnostic validity. This multi-step algorithm, requiring diagnostic codes, symptom codes, objective laryngeal evaluation, and prescription continuity, was adopted to reduce misclassification inherent to code-only definitions.

### 2.3. Study Population

A total of 6550 individuals with a history of reflux disease were initially screened from the 57,559 eligible participants. Among them, we excluded males (*n* = 2924) and individuals younger than 20 years (*n* = 105), yielding 3521 female reflux patients. After excluding those with missing data on female reproductive history (*n* = 2174), 1347 females were included in the final LPR cohort. For the control group, we selected 35,094 women who did not meet the operational definition of LPR at any time during the study period. After excluding males (*n* = 8874) and individuals younger than 20 years (*n* = 2517), 23,703 women were eligible as potential controls.

### 2.4. Propensity Score Matching

Propensity score matching was performed at a 1:1 ratio to minimize selection bias between the LPR cohort and the control group. Matching variables included age, household income level, and educational status. Age, household income, and education were selected for matching because they are strong determinants of both healthcare-seeking behavior (and thus LPR detection) and reproductive timing, and do not lie on the causal pathway. BMI, smoking, alcohol, hypertension, and diabetes were not matched—to avoid over-matching and preserve sample size—but were instead adjusted for in the regression models. After matching, a total of 1347 women without reflux disease were selected as controls and compared with the 1347 women in the LPR cohort.

### 2.5. Statistical Analysis

Statistical analyses were performed using R software (v4.3.1; R Foundation for Statistical Computing, Vienna, Austria). Categorical variables were presented as numbers and percentages and compared using the chi-square test or Fisher’s exact test, as appropriate. Continuous variables were expressed as the mean ± standard deviation and compared using the independent *t*-test. Propensity score matching (1:1 nearest neighbor matching without replacement) was conducted based on age, household income, and education level using the “MatchIt” package (v4.7.2) in R. Post-matching balance was assessed using standardized mean differences (SMDs), with |SMD| < 0.10 indicating negligible imbalance. Because propensity-score matching yields a balanced analytic cohort rather than fixed matched sets, associations between reproductive factors and the risk of LPR were estimated using multivariable unconditional logistic regression, an approach widely used for propensity-score-matched analyses. Reproductive exposures were additionally modeled as continuous variables (Supplementary Table S1). Cut-offs for first delivery age (≥27 years), menarche-to-first-birth interval (≥17 years), and breastfeeding duration (<12 months) were defined based on the sample distribution and clinical interpretability; continuous analyses were performed in parallel to confirm robustness. Three multivariable models were constructed: Model 1 was adjusted for age and body mass index (BMI); Model 2 was additionally adjusted for hypertension and diabetes mellitus; and Model 3 was further adjusted for smoking status and alcohol consumption. Odds ratios (ORs) and 95% confidence intervals (CIs) were calculated. To account for multiple comparisons, *p*-values for the primary exposures were adjusted using the Benjamini–Hochberg false-discovery-rate procedure. A *p*-value < 0.05 was considered statistically significant.

### 2.6. Ethical Considerations

This study was conducted in accordance with the Declaration of Helsinki. All data were anonymized prior to analysis, and individual informed consent was waived due to the retrospective nature of the study. The study protocol was approved by the Institutional Review Board of Hallym University (IRB No. 2023-03-010) and by the Korea Disease Control and Prevention Agency.

### 2.7. Data Availability Statements

All data were obtained from the integrated dataset derived from the Korean National Health Insurance Service and the Korea Disease Control and Prevention Agency and are available at https://hcdl.mohw.go.kr/.

## 3. Results

### 3.1. Baseline Characteristics

**Figure 1:**
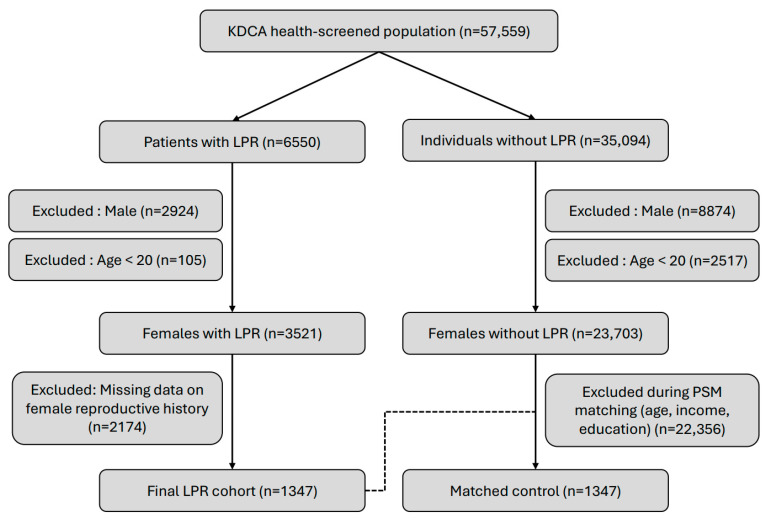
Flow diagram of participant selection. Female participants were selected from the Korea Disease Control and Prevention Agency (KDCA) health-screened population after applying exclusion criteria and propensity score matching (1:1) for age, income, and education. The dashed line indicates the propensity score matching process between the final LPR cohort and the matched control group. LPR, laryngopharyngeal reflux; PSM, propensity score matching; KDCA, Korea Disease Control and Prevention Agency; *n*, number.

After propensity score matching, a total of 2694 female participants were included in the analysis, comprising 1347 patients with LPR and 1347 matched controls without LPR (Figure 1). The baseline demographic and clinical characteristics of the study population are presented in Table 1. There were no significant differences in baseline demographic and clinical characteristics between the two groups (all standardized mean differences < 0.10; Table 1). The prevalence of current alcohol consumption and smoking status was comparable between LPR patients and controls (*p* = 0.625 and *p* = 0.961, respectively). The proportions of participants with hypertension and diabetes mellitus were also similar between the two groups (*p* = 0.651 and *p* = 0.594, respectively). In addition, no significant differences were observed in muscle exercise frequency, education level, or household income level between groups.

### 3.2. Comparison of Female Reproductive Factors

Female reproductive characteristics are summarized in Table 2. The history of pregnancy and number of pregnancies did not differ significantly between the LPR and control groups (*p* = 0.562 and *p* = 0.267, respectively). However, women with LPR had a significantly higher mean age at first delivery compared to controls (25.2 ± 4.1 vs. 24.7 ± 3.9 years, *p* = 0.016). In subgroup analysis, first delivery at age ≥ 27 years was more common in the LPR group than in the control group (35.9% vs. 29.8%, *p* = 0.008). Furthermore, the interval between menarche and first birth was significantly longer in the LPR group (10.4 ± 5.1 years vs. 9.9 ± 4.9 years, *p* = 0.038), and a greater proportion of women in the LPR group had a menarche-to-first-birth interval ≥ 17 years (13.0% vs. 8.9%, *p* = 0.008). Breastfeeding history did not differ significantly; however, breastfeeding duration < 12 months was more frequent among women with LPR (55.4% vs. 50.4%, *p* = 0.012).

### 3.3. Association Between Reproductive Factors and LPR

Table 3 presents the logistic regression analysis of reproductive factors associated with LPR. Unadjusted analysis showed that first delivery at age ≥ 27 years (OR 1.32, 95% CI 1.08–1.61), menarche-to-first-birth interval ≥ 17 years (OR 1.53, 95% CI 1.13–2.08), and breastfeeding duration < 12 months (OR 1.22, 95% CI 1.05–1.43) were significantly associated with an increased risk of LPR. These associations remained significant after adjusting for potential confounders in Model 1 (age and BMI), Model 2 (Model 1 + hypertension and diabetes mellitus), and Model 3 (Model 2 + smoking and alcohol consumption). In the fully adjusted model, first delivery at age ≥ 27 years (OR 1.33, 95% CI 1.08–1.63), a menarche-to-first-birth interval ≥ 17 years (OR 1.56, 95% CI 1.15–2.15), and breastfeeding < 12 months (OR 1.26, 95% CI 1.04–1.53) remained independently associated with LPR. All three associations remained significant after Benjamini–Hochberg correction (adjusted *p* = 0.012–0.016). Consistent associations were observed when reproductive factors were modeled continuously (Supplementary Table S1).

## 4. Discussion

This nested case–control study demonstrated that female reproductive factors that may reflect cumulative hormonal exposure were significantly associated with LPR. Specifically, first delivery at age ≥ 27 years, a menarche-to-first-birth interval ≥ 17 years, and breastfeeding < 12 months were independently associated with an increased risk of LPR in women, even after adjusting for socioeconomic, lifestyle, and metabolic factors. To our knowledge, this is the first large-scale study to evaluate the relationship between reproductive history and LPR using a nationally representative cohort.

Our findings are consistent with a substantial body of literature demonstrating a link between female reproductive and hormonal factors and GERD. Several studies have consistently demonstrated that female reproductive and hormonal factors play a significant role in the pathophysiology of GERD. Pregnancy, menopause, and the use of hormone replacement therapy have each been associated with an increased risk or exacerbation of GERD symptoms [9]. During pregnancy, elevated levels of estrogen and progesterone reduce the tone of the lower esophageal sphincter and delay gastrointestinal motility, resulting in reflux symptoms in up to 80% of women [10,11]. Similarly, in postmenopausal women, hormonal fluctuations modify esophageal motility and mucosal protection [12]. While the menopausal decline in estrogen may weaken sphincter integrity, exogenous hormone replacement has paradoxically been shown to increase GERD prevalence, possibly due to nitric oxide-mediated smooth muscle relaxation of the lower esophageal sphincter [13]. However, while the association between hormonal factors and GERD has been increasingly recognized, evidence specific to LPR remains scarce.

Beyond statistical associations, these findings provide valuable insights into sex-related susceptibility to reflux disease. Prior studies have demonstrated that LPR involves distinct pathophysiological processes compared with GERD. Unlike GERD, which predominantly causes esophageal mucosal injury through prolonged acid exposure, LPR often results from intermittent upright reflux and exposure of the laryngeal and pharyngeal mucosa to small amounts of gastric contents, including acid, pepsin, and bile salts [14]. These elements may trigger neurogenic inflammation, epithelial permeability changes, and heightened mucosal sensitivity rather than overt tissue erosion [15]. Consequently, women with hormonal variability or diminished mucosal defense, such as during the perimenopausal period, may exhibit a lower threshold for symptom onset even in the presence of nonacid or weakly acidic reflux episodes [16].

The following mechanistic interpretations are speculative and hypothesis-generating, as no hormonal, menopausal, or hormone-therapy data were available in this study. Our results align with emerging evidence that endocrine and mucosal factors interact to shape reflux phenotypes. Previous investigations have suggested that sex hormones influence vagal tone, smooth muscle relaxation of the upper esophageal sphincter, and sensory modulation of laryngopharyngeal mucosa [17]. Furthermore, pepsin and bile reflux components can penetrate epithelial cells, activate proinflammatory pathways, and sensitize local afferent fibers [18]. These mechanisms provide a biologically plausible explanation for the observed correlation between reproductive history and LPR risk, particularly in women with breastfeeding < 12 months or first delivery at age ≥ 27 years—conditions reflecting prolonged exposure to fluctuating estrogen and progesterone levels. The observed associations were modest (odds ratios 1.2–1.6) and should be regarded as hypothesis-generating rather than indicative of strong or causal effects.

Clinically, these findings suggest that reproductive history may be considered in the evaluation and management of LPR. Women presenting with chronic throat symptoms during high-hormone states may warrant further evaluation, a possibility that should be examined in future prospective studies, although this study does not establish causal relationships. Recognition of hormonal influences could also inform public health strategies and patient counseling, especially in populations with increasing age at first childbirth.

This study has several limitations. First, the diagnosis of LPR relied on administrative diagnostic and prescription codes rather than objective pH-impedance measurements or validated symptom indices (RSI/RFS), which may have led to misclassification. Second, more than half of the eligible women with LPR were excluded because of missing reproductive history data; because the data-access period has closed, covariates of the excluded individuals could not be re-extracted, precluding a formal comparison between included and excluded participants. Potential selection bias therefore cannot be excluded, and generalizability should be interpreted cautiously. Third, several potentially relevant variables—including menopausal status, hormone-replacement therapy, and oral-contraceptive use—were unavailable; because age (the principal determinant of menopausal status) was both matched and adjusted, additional conditioning on menopausal status would risk over-adjustment and reduced statistical power, so these remain residual confounders. Fourth, dichotomizing continuous exposures may lose information, although analyses using the continuous variables yielded consistent results. Fifth, formal multicollinearity and interaction testing were not feasible within the data-access constraints. Finally, the matched, claims-based design precludes firm temporal or causal inference, and residual confounding inherent to observational studies cannot be fully excluded. Despite these limitations, the large, nationally representative sample and the consistency of estimates across sequentially adjusted and continuous models support the robustness of our findings.

In summary, this study highlights the epidemiologic link between female reproductive history and LPR risk and suggests that hormonal and mucosal mechanisms may contribute to reflux vulnerability. Future prospective studies integrating hormonal profiling, impedance–pH analysis, and mucosal biomarker evaluation are warranted to elucidate the endocrine–reflux interface in greater depth.

## 5. Conclusions

Female reproductive factors potentially reflecting cumulative hormonal exposure were significantly associated with laryngopharyngeal reflux in this nested case–control study. First delivery at age ≥ 27 years, a menarche-to-first-birth interval ≥ 17 years, and breastfeeding < 12 months were independently associated with LPR. These findings suggest that reproductive history may be associated with reflux susceptibility in women and may warrant consideration in clinical evaluation.

## Figures and Tables

**Table 1:** Baseline demographic and clinical characteristics of the study participants after propensity score matching.

	Controls	Reflux Patients	*p*-Value	SMD
	(*N* = 1347)	(*N* = 1347)		
Age (y)	53.9 ± 15.0	53.9 ± 15.0	0.997	0.00
Height (cm)	155.6 ± 19.4	155.8 ± 19.4	0.688	0.01
Weight (kg)	56.5 ± 17.5	56.5 ± 17.4	0.900	0.00
Waist circumference (cm)	76.8 ± 13.8	76.7 ± 13.9	0.860	0.01
BMI, kg/m^2^	22.6 ± 4.1	22.5 ± 4.1	0.569	0.02
Current alcohol use			0.625	0.02
- Yes	896 (66.5%)	884 (65.6%)		
- No	451 (33.5%)	463 (34.4%)		
Current smoking			0.961	0.00
- Yes	261 (19.4%)	260 (19.3%)		
- No	1086 (80.6%)	1087 (80.7%)		
Muscle exercise (>once per week)			0.841	0.01
- Yes	482 (35.8%)	477 (35.4%)		
- No	865 (64.2%)	870 (64.6%)		
Hypertension			0.651	0.02
- Yes	236 (17.5%)	245 (18.2%)		
- No	1111 (82.5%)	1102 (81.8%)		
Diabetes mellitus			0.594	0.02
- Yes	89 (6.6%)	96 (7.1%)		
- No	1258 (93.4%)	1251 (92.9%)		
Residual Area			0.962	0.00
- Urban	1062 (78.8%)	1063 (78.9%)		
- Rural	285 (21.2%)	284 (21.1%)		
Education level			0.999	0.04
- Elementary school	535 (39.8%)	519 (39.7%)		
- Middle school	154 (11.4%)	133 (10.2%)		
- High school	368 (27.3%)	372 (28.4%)		
- Colleges	290 (21.5%)	284 (21.7%)		
Household income level			1.000	0.00
- Very low	307 (22.8%)	307 (22.8%)		
- Low	216 (16.0%)	216 (16.0%)		
- Medium	267 (19.8%)	267 (19.8%)		
- High	287 (21.3%)	287 (21.3%)		
- Very high	270 (20.0%)	270 (20.0%)		

Continuous variables are the mean ± SD; categorical variables are No. (%). SMD, standardized mean difference (|SMD| < 0.10 indicates negligible imbalance). All covariates were well balanced after 1:1 propensity score matching. Age, household income, and education were matching variables; their distributions are balanced by design. BMI, body mass index.

**Table 2:** Comparison of female reproductive factors between participants with and without laryngopharyngeal reflux.

	Controls	Reflux Patients	*p*
	(*N* = 1347)	(*N* = 1347)	
Pregnancy history			0.562
- Yes	900 (70.2%)	907 (69.1%)	
- No	382 (29.8%)	406 (30.9%)	
Number of Pregnancies (*n*)	2.8 ± 2.7	2.7 ± 2.7	0.267
Delivery history			0.728
- Yes	879 (68.7%)	893 (68.0%)	
- No	401 (31.3%)	421 (32.0%)	
Age at first delivery (years)	24.7 ± 3.9	25.2 ± 4.1	0.016 *
First delivery age group			0.008 *
- First delivery < 27 years	614 (70.2%)	569 (64.1%)	
- First delivery ≥ 27 years	261 (29.8%)	319 (35.9%)	
Menarche age (y)	14.4 ± 2.3	14.4 ± 2.2	0.462
Menarche-to-first-birth interval (years)	9.9 ± 4.9	10.4 ± 5.1	0.038 *
Menarche-to-first-birth interval group			0.008 *
- Menarche-to-first-birth interval < 17 years	788 (91.1%)	764 (87.0%)	
- Menarche-to-first-birth interval ≥ 17 years	77 (8.9%)	114 (13.0%)	
Breastfeeding history			0.839
- Yes	775 (60.5%)	787 (60.0%)	
- No	507 (39.5%)	525 (40.0%)	
Breastfeeding duration (months)	25.7 ± 39.9	23.5 ± 39.1	0.16
Breastfeeding duration group			0.012 *
- Breastfeeding duration < 12 months	641 (50.4%)	723 (55.4%)	
- Breastfeeding duration ≥ 12 months	631 (49.6%)	582 (44.6%)	

Mean ± SD. * *p* < 0.05. Data are expressed as No. (%) of patients.

**Table 3:** Association between reproductive factors and laryngopharyngeal reflux (Logistic regression analysis).

Reproductive Factor	Unadjusted	Model 1	Model 2	Model 3
First delivery age ≥ 27 y	1.32 (1.08–1.61)	1.31 (1.07–1.61)	1.33 (1.08–1.63)	1.33 (1.08–1.63)
	*p* = 0.006	*p* = 0.010	*p* = 0.007	*p* = 0.007
Menarche-to-first-birth interval ≥ 17 y	1.53 (1.13–2.08)	1.54 (1.13–2.10)	1.54 (1.13–2.11)	1.56 (1.15–2.15)
	*p* = 0.006	*p* = 0.006	*p* = 0.007	*p* = 0.005
Breastfeeding < 12 months	1.22 (1.05–1.43)	1.22 (1.03–1.44)	1.23 (1.03–1.47)	1.26 (1.04–1.53)
	*p* = 0.012	*p* = 0.020	*p* = 0.023	*p* = 0.019

Values are odds ratios (95% confidence intervals) with *p*-values. Model 1: adjusted for age and BMI; Model 2: Model 1 + hypertension and diabetes mellitus; Model 3: Model 2 + smoking and alcohol consumption. After Benjamini–Hochberg correction for multiple testing (Model 3), all three associations remained significant (FDR-adjusted *p*: first delivery ≥ 27 y, 0.012; menarche-to-first-birth ≥ 17 y, 0.015; breastfeeding < 12 months, 0.016). BMI, body mass index; FDR, false discovery rate.

## Data Availability

The data that support the findings of this study were obtained from the integrated database of the Korean National Health Insurance Service (NHIS) and the Korea Disease Control and Prevention Agency (KDCA). Restrictions apply to the availability of these data, which were used under license for the current study and are therefore not publicly available. Data are available from the corresponding author upon reasonable request and with permission from the NHIS and KDCA.

## Supplementary Materials

The following supporting information can be downloaded at: https://www.mdpi.com/article/10.3390/jcm15135279/s1, Table S1: Reproductive factors modeled as continuous variables (sensitivity analysis). Table S2: Additional reproductive variables examined (not significantly associated).
