# Supplementary material for: Association Between Female Reproductive Factors and Laryngopharyngeal Reflux: A National Population-Based Study
Source: J Clin Med. 2026 Jul 6;15(13):5279. doi: 10.3390/jcm15135279 (PMC13362644; doi:10.3390/jcm15135279)
Supplement: Supplementary file 1 [file jcm-15-05279-s001.zip › jcm-4379338-supplementary.pdf]

Supplementary Table S1. Reproductive factors modeled as continuous variables (sensitivity analysis).

Reproductive factor (continuous)	Unadjusted OR (95% CI), p	Adjusted OR (Model 3) (95% CI), p
Age at first delivery (per 1-year increase)	1.03 (1.01–1.05), p = 0.016	1.03 (1.01–1.06), p = 0.014
Menarche-to-first-birth interval (per 1-year increase)	1.02 (1.00–1.04), p = 0.039	1.02 (1.00–1.04), p = 0.031
No. of breastfed children (per 1-child increase)	0.95 (0.91–1.00), p = 0.032	0.95 (0.90–1.00), p = 0.056

Each reproductive exposure was modeled as a continuous variable. The associations are consistent in direction and significance with the dichotomized analyses in Table 3, indicating the findings are not artifacts of the chosen cut-offs. Model 3 adjusted for age, BMI, hypertension, diabetes mellitus, smoking, and alcohol consumption. OR, odds ratio; CI, confidence interval.

Supplementary Table S2. Additional reproductive variables examined (not significantly associated).

Variable	Normal Control	Reflux Patients	p
Pregnancy experience (yes)	70.20%	69.10%	0.562
No. of pregnancies (mean)	2.8 ± 2.7	2.7 ± 2.7	0.267
Delivery experience (yes)	68.70%	68.00%	0.728
Last delivery age ≥35 y	14.90%	15.80%	0.649
Menarche age (y)	14.4 ± 2.3	14.4 ± 2.2	0.462
Breastfeeding experience (yes)	60.50%	60.00%	0.839
Breast cancer diagnosis (yes)	0.50%	0.30%	0.432
Cervical cancer diagnosis (yes)	0.50%	0.30%	0.415

Presented to document that the full set of available reproductive and gynecologic variables was examined. Values are No. (%) or mean ± SD.
